# Immunoliposomes doubly targeted to transferrin receptor and to α-synuclein

**DOI:** 10.4155/fso.15.71

**Published:** 2015-09-10

**Authors:** Joana A Loureiro, Bárbara Gomes, Manuel AN Coelho, Maria do Carmo Pereira, Sandra Rocha

**Affiliations:** 1 LEPABE, Department of Chemical Engineering, Faculty of Engineering of the University of Porto, 4200-465 Porto, Portugal; 2 Department of Biology & Biological Engineering, Chalmers University of Technology, Gothenburg SE-41296, Sweden

**Keywords:** α-synuclein, blood–brain barrier, drug delivery, dual targeting, immunoliposomes, peptidomimetic monoclonal antibodies, transferrin receptor

## Abstract

**Aim:**

The present study was designed to test the cellular uptake of PEGylated liposomes targeted to transferrin receptor and to α-synuclein by a cell model of the blood–brain barrier (BBB).

**Materials & methods:**

PEGylated immunoliposomes were prepared with anti-transferrin receptor OX26 and anti-α-synuclein LB509 antibodies to overcome the BBB in Parkinson's disease.

**Results:**

The doubly targeted immunoliposomes bind to transferrin receptor and to α-synuclein protein, as assessed by ELISA assays. We establish that 40% of an encapsulated tested drug (epigallocatechin-3-gallate) is released in a time frame of 44 h, which is reasonable for sustained release. The cellular uptake of doubly targeted immunoliposomes in cultured brain endothelial cells hCMEC/D3 was two-times more efficient than that of PEGylated liposomes.

**Conclusion:**

Immunoliposomes targeted to BBB receptors and to α-synuclein could potentially enable the transport of drugs across the BBB and reach one of the drug targets in Parkinson's disease.

## Abstract (lay abstract)

**Lay abstract:** The blood–brain barrier (BBB) prevents the distribution of drugs into the brain, making the development of new treatments for brain disorders such as Parkinson's disease difficult. This is due to the presence of tight cell–cell junctions within the brain capillary endothelium. Nanocarriers that transport drugs across the BBB enable noninvasive modes of drug delivery (e.g., oral, systemic routes) to the brain. In the present study, we developed vesicles targeted with antibodies to BBB receptors and to a biological target of Parkinson's disease. This technology, known as Trojan horse technology, uses endogenous molecules that are able to cross the BBB through receptors present in the brain capillary endothelium.

**Figure 1.**
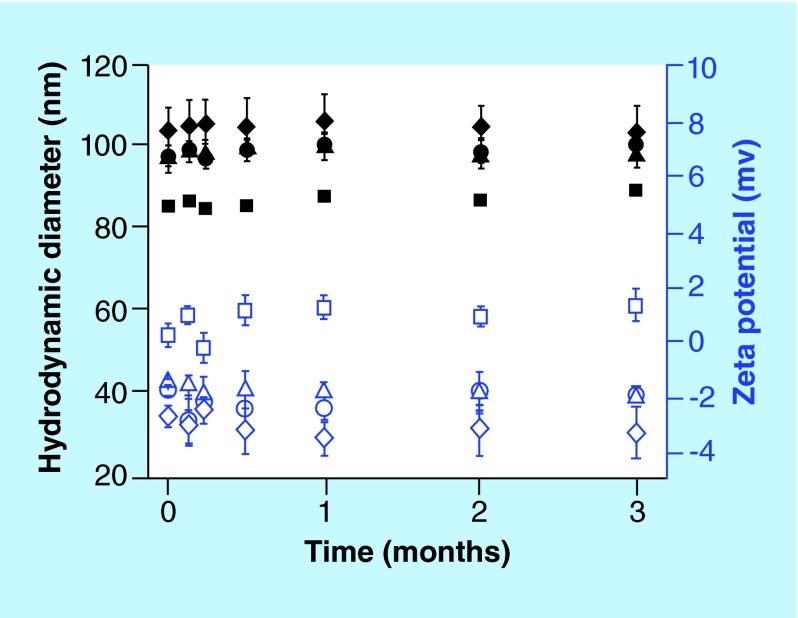
**Physical stability of immunoliposomes.** Variation of the hydrodynamic diameter (black symbols) and the zeta potential (blue open symbols) of liposomes over 3 months: PEGylated liposomes (▪), PEGylated liposomes with anti-α-synuclein antibody LB509MAb (•), PEGylated liposomes with anti-transferrin receptor antibody OX26MAb (triangle) and PEGylated liposomes with OX26MAb and LB509MAb (♦). Error bars are standard deviations of mean (n = 3).

**Figure 2.**
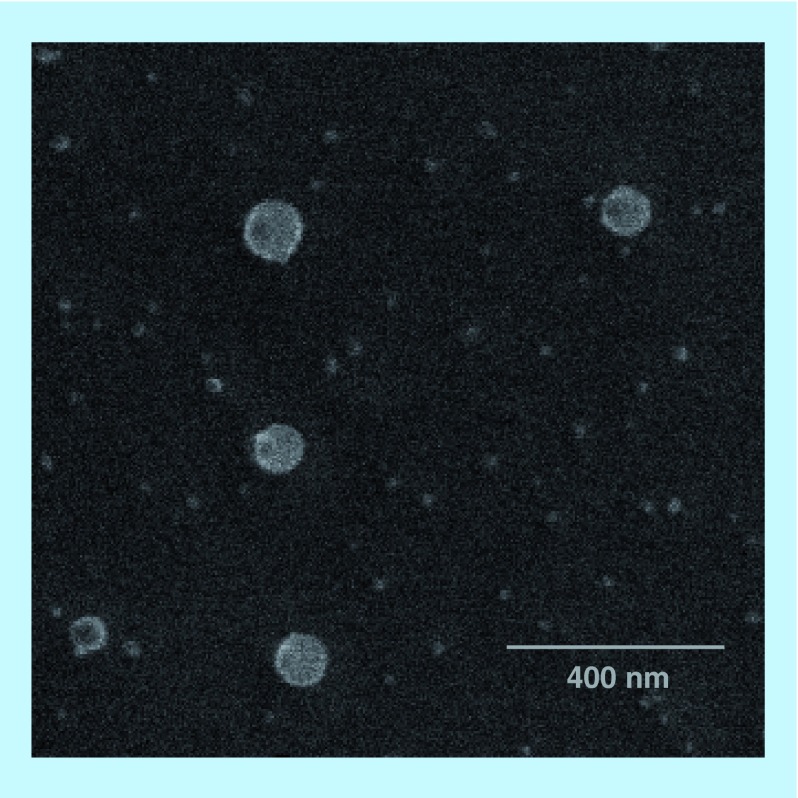
**Cryo-scanning electron micrograph of PEGylated liposomes.**

**Figure 3.**
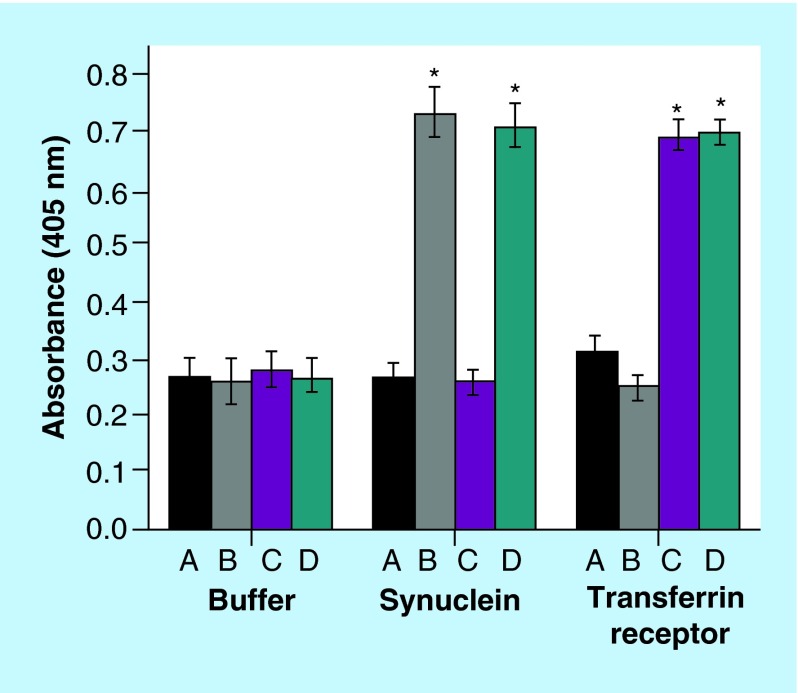
**Binding affinity.** Binding of immunoliposomes to target proteins α-synuclein and transferrin receptor assessed by ELISA. A: PEGylated liposomes; B: PEGylated liposomes with anti-α-synuclein antibody LB509MAb; C: PEGylated liposomes with anti-transferrin receptor antibody OX26MAb; D: PEGylated liposomes with OX26MAb and LB509MAb. Phosphate saline buffer was used as a control. Error bars are standard deviations of mean (n = 3). *Statistically significant from A in each group (p < 0.001).

**Figure 4.**
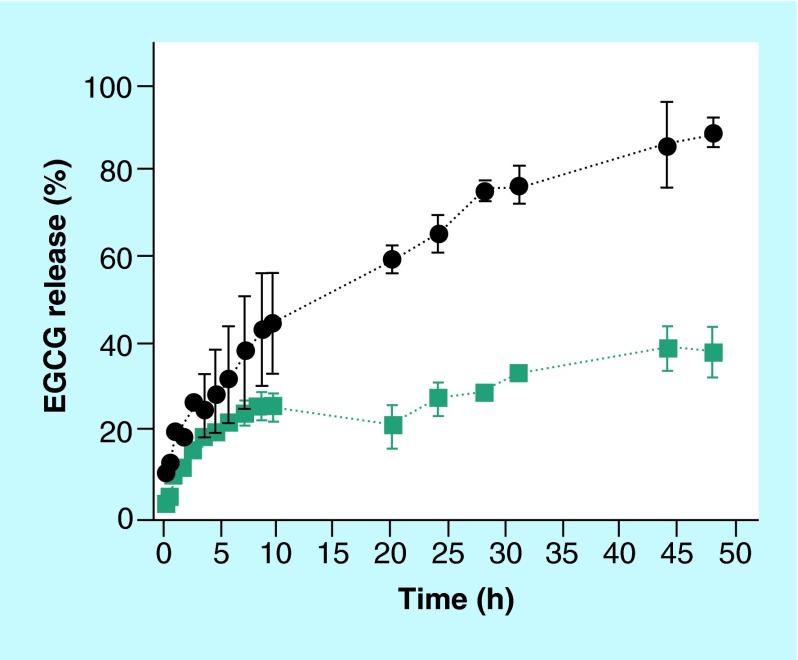
***In vitro* epigallocatechin-3-gallate release profile from immunoliposomes.** The liposomes are coupled with anti-transferrin receptor antibody OX26MAb and anti-α-synuclein antibody LB509MAb and the drug release was studied using dialysis membranes (molecular weight cutoff: 100 kDa) (dark cyan squares). The permeation of nonencapsulated drug through the membrane is shown as a control (black circles). Error bars are standard deviations of mean (n = 3). EGCG: Epigallocatechin-3-gallate.

**Figure 5.**
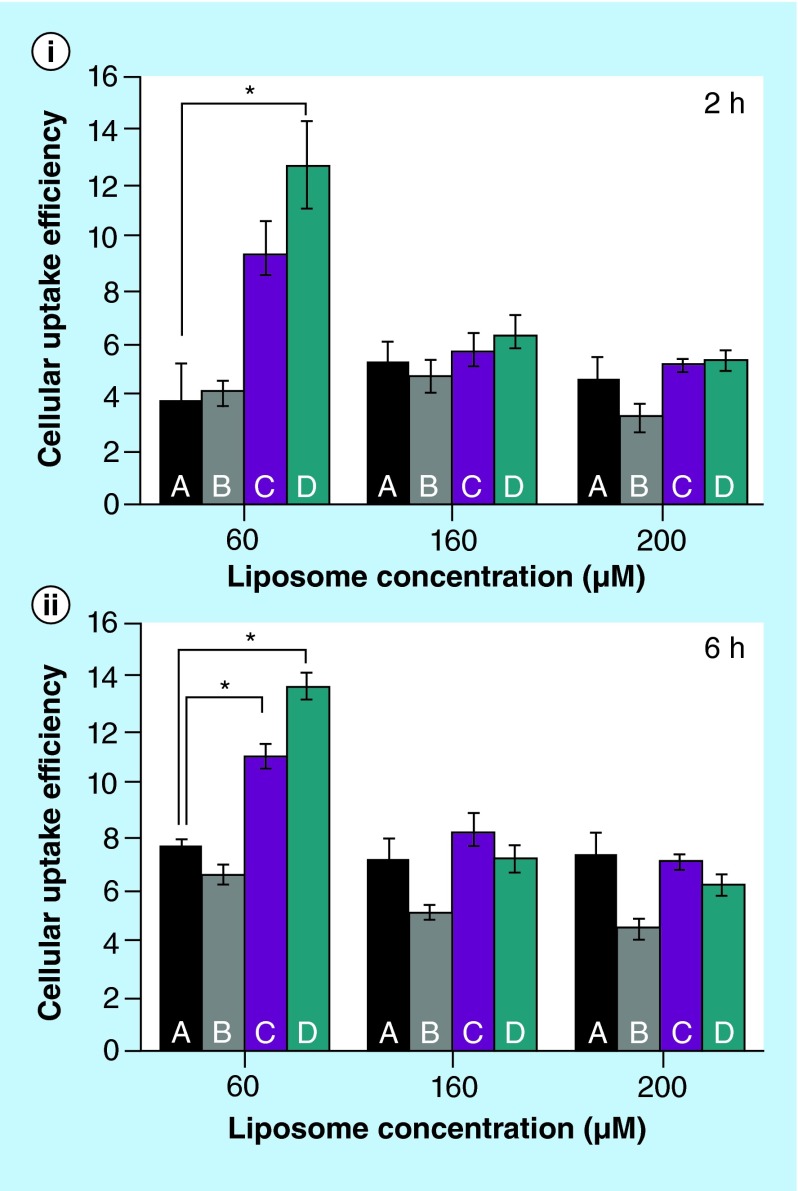
**Cellular uptake of liposomes in hCMEC/D3 cells as a function of concentration and of incubation time.** **(i)** 2 h and **(ii)** 6 h. A: PEGylated liposomes; B: PEGylated liposomes with anti-α-synuclein antibody LB509MAb; C: PEGylated liposomes with anti-transferrin receptor antibody OX26MAb; D: PEGylated liposomes with OX26MAb and LB509MAb. The uptake is measured as mean fluorescence. The concentration relates to the phospholipid content. Error bars are standard deviations of mean values (n = 3). *p < 0.04.

**Figure 6.**
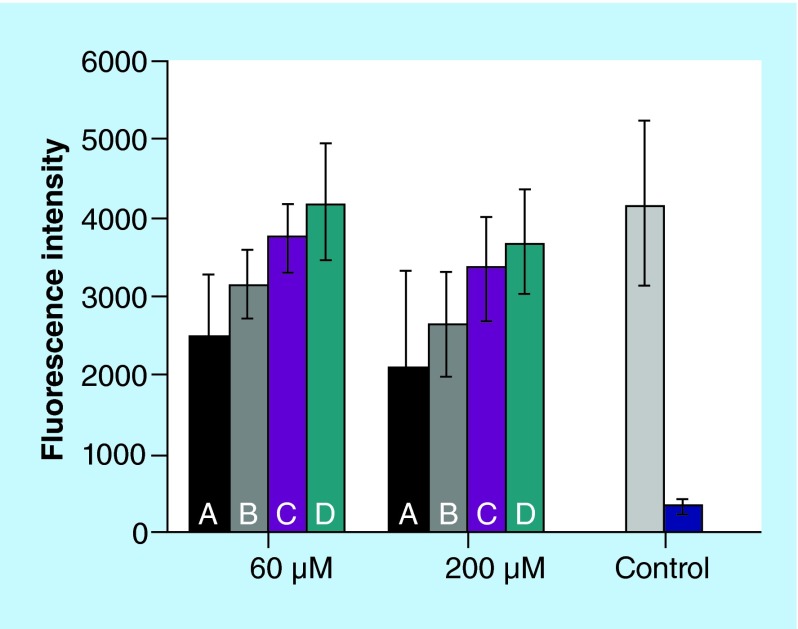
**Cytotoxicity of immunoliposomes.** Metabolic activity of hCMEC/D3 cells incubated for 2 h with liposomes. A: PEGylated liposomes; B: PEGylated liposomes with anti-α-synuclein antibody LB509MAb; C: PEGylated liposomes with anti-transferrin receptor antibody OX26MAb; D: PEGylated liposomes with OX26MAb and LB509MAb. Control corresponds to cells incubated only with buffer (light gray) or with Triton (blue). Error bars are standard deviations of mean (n = 3).

Parkinson's disease (PD), a neurodegenerative disorder for which there is no cure, affects nearly seven million people worldwide. Clinically, the disease evolves from subtle nonspecific nonmotor manifestations to advanced stages with severe motor (dyskinesias and rigidity) and cognitive impairments [1,2]. The pathological hallmarks of the disease are characterized by a prominent loss of dopamine-producing neurons in the substantia nigra (SN), which causes dopamine reduction in the striatum and the presence of neuronal cytoplasmic inclusions known as Lewy bodies [3]. Since Lewy bodies are rich in aggregated α-synuclein, this protein is proposed to be a key factor in the pathogenesis of PD [4]. α-synuclein is a natively unordered, soluble protein that spontaneously self-assembles into insoluble aggregates or amyloid fibrils [5]. The conformational changes of the protein lead to the formation of insoluble neurotoxic aggregates [6,7]. The inhibition of α-synuclein aggregation has become a valid therapeutic target in PD.

For noninvasive administration, drugs developed for the treatment of PD or other neurodegenerative disorders need to cross the blood–brain barrier (BBB). This barrier is the cerebral microvascular endothelium, which has tight cell–cell junctions and few alternate transport pathways (e.g., decreased pinocytotic activity and significantly decreased number of intracellular fenestrae). The targeting of liposomes across the BBB with receptor-specific peptidomimetic monoclonal antibodies (MAbs) is a known technology that enables the delivery of drugs noninvasively to the brain [8]. The drugs can be encapsulated in the interior of liposomes that are targeted to the BBB receptors with MAbs. This approach allows higher carrying capacities of the MAbs than the individually conjugation of drugs to MAbs.

We prepared PEGylated liposomes doubly targeted with the OX26MAb to the rat transferrin receptor and the LB509MAb to the α-synuclein. The flavonoid epigallocatechin-3-gallate (EGCG) was encapsulated in the interior of the liposomes as a model drug. EGCG is a natural antioxidant capable of inhibiting α-synuclein toxicity [9]. It inhibits the protein aggregation and the α-synuclein oligomers’ ability of inducing cytotoxicity in brain cells [9,10]. The EGCG bioavailability is low, only 0.1% after administration of tea polyphenols, and its systemic clearance is high, rendering EGCG use for the treatment of CNS disorders very limited [11]. Although the BBB targeting with MAbs against endogenous BBB receptors is a well-studied technology [12], the use of two different MAbs to target liposomes to the brain is less developed. The present study was designed to test the cellular uptake of doubly targeted PEGylated liposomes to transferrin receptor and to α-synuclein by a cell model of the BBB.

## Materials & methods

### Formulation of liposomes

All lipids were purchased from Avanti Polar Lipids. The liposomes were prepared by the lipid film hydration method. DSPC (1,2-distearoyl-*sn*-glycero-3-phosphocholine), Chol (cholesterol ovine wool), DSPE-PEG_2000_ (1,2-distearoyl-*sn*-glycero-3-phosphoethanolamine-*N*-[amino(polyethylene glycol)-2000] ammonium salt), DSPE-PEG_2000_-maleimide (1,2-distearoyl-*sn*-glycero-3-phosphoethanolamine-*N*-[maleimide(polyethylene glycol)-2000] ammonium salt), DSPE-PEG_2000_-biotin (1,2-distearoyl-*sn*-glycero-3-phosphoethanolamine-*N*-[biotin(polyethylene glycol)-2000] ammonium salt) and LissRhod-PE (1,2-dipalmitoyl-*sn*-glycero-3-phosphoethanolamine-*N*-(lissamine rhodamine B sulfonyl) ammonium salt, Avanti Polar Lipids) were dissolved in chloroform at a molar ratio of 52:45:3:0.015:0.015:0.01. The solvent was evaporated using a nitrogen stream in a rotary evaporator and the resultant dried lipid film was dispersed in PBS buffer, pH 7.4 (phosphate buffered saline, 10 mM phosphate buffer, 2.7 mM potassium chloride and 137 mM sodium chloride, Sigma-Aldrich) with a final total lipid concentration of 0.80 mM. The liposome suspension was vortexed, frozen-thawed and then extruded through polycarbonate filters with pore diameters of 100 nm and 50 nm at least ten-times using a Mini Extruder (Avanti Polar Lipids). The fluorescence intensity of each type of liposomes was measured with a Synergy 2 Multi-Mode Microplate Reader (BioTek Instruments Inc., with excitation filter 528/20 nm and emission filter 580/50 nm) and the concentration adjusted to the same value.

### Conjugation of antibodies

The MAb against the transferrin BBB receptor (OX26MAb) was obtained from AbD Serotec and the MAb against α-synuclein (LB509MAb) was purchased from Abcam. Covalent coupling methods for attaching the antibodies at the PEG terminus were based on functionalized PEG with a chemically reactive end-group, the PEG-maleimide (thiol reactive) and a PEG-biotin (the biotin–streptavidin method). For the maleimide-MAb conjugation, the MAb was activated by a 20-times molar excess of Traut's reagent (2-iminothiolane hydrochloride, Sigma-Aldrich). A drop of EDTA (ethylenediaminetetraacetic acid, Sigma-Aldrich) 0.28 M was added to prevent metal-catalyzed oxidation of sulfhydryl groups [13]. The unreacted complex EDTA/2-iminothiolane was removed by size exclusion chromatography using a Sephadex column PD-Mini Trap G25 (GE Healthcare) [14]. It has been previously shown that thiolation of MAb does not interfere with their binding site [15]. Since the maleimide group slowly hydrolyzes in aqueous solution, it is essential to perform the antibody conjugation immediately after the preparation of the liposomes. The second MAb was first coupled to a biotin molecule using the EZ-Link microsulfo-NHS-LC-biotin (Thermo Scientific) kit. The MAb was linked to the functionalized PEG-biotin through streptavidin (from *Streptomyces avidinii*, Sigma-Aldrich) at a molar ratio of 1:1. Both antibodies were added to the liposomes at a molar ratio of 1:1 between antibodies and functionalized PEG. The liposomes and the antibodies were incubated at room temperature (RT) for 1 h and then at 5°C for 8 h.

### Liposome characterization

The hydrodynamic diameter and zeta potential of the liposomes were analyzed using a Zetasizer Nano ZS (Malvern Instruments Ltd.). Size measurements are based on photon correlation spectroscopy and zeta potential is based on laser Doppler velocimetry. PEGylated liposomes were also characterized by cryo-scanning electron microscopy (cryo-SEM) using a JEOL JSM 6301F/Oxford INCA Energy 350. The images were acquired with a Gatan Alto 2500.

### Enzyme-linked immunosorbent assay for immunoliposomes

The functional activity of the antibodies conjugated to the liposomes was analyzed by the enzyme-linked immunosorbent assay (ELISA). The surface of 96-well plates (flat-bottom Nunc MaxiSorp^®^) was coated with either transferrin receptor (Abcam) or α-synuclein for 1 h at 37°C. After blocking with bovine serum albumin (BSA), the immunoliposomes were added to each well, followed by a washing step. The secondary antibody conjugated with peroxidase (Goat anti-Mouse IgG [H+L] Cross Adsorbed Secondary Antibody, HRP conjugate, Thermo Scientific-Pierce Antibodies) was then allowed to react for 45 min at RT. To reveal the presence of the antibodies, a citrate solution was used with citric acid (Sigma-Aldrich), ABTS (2,2′-azino-bis(3-ethylbenzothiazoline-6-sulfonic acid) diammonium salt, Sigma-Aldrich) and H_2_O_2_ (hydrogen peroxide solution, Sigma-Aldrich). The color intensity was measured by spectrometry using a spectrometer (Synergy 2 Multi-Mode Microplate Reader, BioTek Instruments Inc.). Liposomes without MAb were used as control. The statistical significance was determined by Student's t-test analysis.

### Catechin encapsulation & release

The loading of liposomes with EGCG (458 Da, Taiyo Kaguku) was performed during the hydration step of the lipid film. The dried lipid film was hydrated with an aqueous solution containing the catechin at molar ratios of 16:1 and 10:1 (phospholipid:peptide). Nonencapsulated catechin was removed by using a Sephadex column PD-Midi Trap G25. For the determination of the encapsulation efficiency, the liposomes were burst with water:ethanol 25:75 (v/v) and the absorption spectrum was measured (Synergy 2 Multi-Mode Microplate Reader, BioTek Instruments Inc.). The encapsulation efficiency was calculated based on the encapsulated fraction and the total EGCG added. The release of EGCG from the liposomes was studied by using a dialysis bag (Float-A-Lyzer G2, CE, 100 kDa, SpectrumLabs). A volume of 2 ml of immunoliposomes was added into the dialysis tube and dialysis was carried out against buffer at 37°C with continuously stirring (200 rpm). Aliquots from the medium outside the dialysis bag were collected at different times. The amount of catechin released from the liposomes was determined by the absorbance at 274 nm. The percentage of the released EGCG at each time point, (Catechin)_t_, is then calculated from Equation 1:
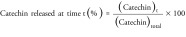


where (Catechin)_total_ is the concentration of catechin added initially to the lipid film.

### Cellular uptake & cytotoxicity assays

Immortalized human cerebral microvascular endothelial cell line (hCMEC/D3) was incubated at 37°C in complete medium (Fetal Bovine Serum ‘Gold’ 5% [PAA, The Cell Culture Company], penicillin/streptomycin 1% [Penicillin, 10,000 units; Streptomycin, 10,000 µg/ml – Invitrogen, Gibco], hydrocortisone 1.4 µM [Sigma-Aldrich], acid ascorbic 5 µg/ml [Sigma-Aldrich], chemically defined lipid concentrate 1/100 [Invitrogen, Gibco], HEPES 10 mM [PAA, The Cell Culture Company], human basic fibroblast growth factor 1 ng/ml [bFGF, Sigma-Aldrich] and endothelial basal medium [EBM-2, Lonza]) in a 96-well plate (Corning) previously coated with Cultrex^®^ Rat Collagen I (R&D Systems, Trevigen). The culture medium was replaced at every 48 h incubation time. Normally, apparent confluence of the monolayers was reached after 6 days (≈6–8 × 10^4^ cells per well). At day 6, the medium was replaced by a solution of Krebs Ringer buffer (KRB) containing different concentrations of the immunoliposomes. KRB is composed by NaCl (sodium chloride p.A., AppliChem), KCl (potassium chloride, AppliChem), H_2_KO_4_P (potassium dihydrogen phosphate p.A., AppliChem), HEPES (hepes puffedran ≥99.5% p.A., ROTH), d-Glucose (d(+)-glucose anhydrous for biochemistry, MERCK), MgCl_2_·6H_2_O (magnesium chloride hexahydrate p.A., AppliChem) and CaCl_2_·2H_2_O (calcium chloride dehydrate p.A., AppliChem). The cells were incubated with the immunoliposomes at 37°C for 2 and 6 h. After removal of the suspension by aspiration, the cells were rinsed at 4°C: three-times with medium, then with a buffer pH 3 (26 mM of sodium citrate, Sigma; 9.2 mM citric acid monohydrated, Grussing; 90.1 mM NaCl and 30 mM KCl) for 5 min followed by KRB. The supernatants were discarded and the cells were lysed with a Triton X-100 1% solution for 1 h at 65°C. The fluorescence intensity was measured with a Synergy 2 Multi-Mode Microplate Reader (BioTek Instruments Inc., with excitation filter 528/20 nm and emission filter 580/50 nm). The initial concentration of liposomes was determined by fluorescence and was within the linear range of detection of the method.

For the cytotoxicity assays, the immunoliposomes were incubated with the cells for 2 h at 37°C. Cells without liposomes and lysed cells by 1% Triton were used as negative and positive controls, respectively. After removal of the immunoliposomes suspension or medium (controls) by aspiration, the cells were incubated with Alamar Blue reagent 40:1 (water:Alamar Blue – v/v) (Thermo Scientific) up to 4 h. The fluorescence was detected with a Synergy 2 Multi-Mode Microplate Reader (BioTek Instruments Inc., excitation 528/20 nm and emission 580/50 nm).

Student's t-test statistical analysis was used to determine statistical significance between cells exposed to PEGylated liposomes (control) and cells exposed to antibody-decorated PEGylated liposomes. A p-value of less than 0.05 was considered statistically significant.

## Results & discussion

### Liposome formulations

We prepared the following systems: A) PEGylated liposomes without antibodies (control); B) PEGylated liposomes with anti-α-synuclein antibody LB509MAb coupled through maleimide; C) PEGylated liposomes with anti-transferrin receptor antibody OX26MAb coupled through streptavidin-biotin system; and D) PEGylated liposomes with both OX26MAb and LB509MAb (OX26MAb-/LB509MAb-PEGylated liposomes).

The hydrodynamic diameter of the liposomes ranges between 86 nm (control liposomes) and 105 nm (liposomes with antibodies) (Table 1 and Figure 1). These results are in accordance with the size observed by cryo-SEM for PEGylated liposomes. The images show spherical liposomes with an average diameter of approximately 100 nm (Figure 2). The variations of the diameter observed for immunoliposomes are related to the size of the antibodies and bioconjugate reagent. The hydrodynamic diameter of immunoliposomes coupled to LB509MAb (formulation B) is about 12 nm larger than that of the control liposomes, which is approximately the diameter of globular MAbs. For the formulation C and D, the diameter is 18–19 nm larger, which might account for the streptavidin-biotin system and MAbs. The addition of MAbs to the liposomes does not significantly affect their zeta potential, even if a small variation to negative values is observed (Table 1).

The size and zeta potential of the immunoliposomes were measured for 3 months to assess their physical stability (Figure 1). There were no significant variations on these parameters, which indicate that the immunoliposomes are stable at least over 3 months [16]. Since aggregation could decrease the cellular uptake efficiency of the immunoliposomes, it is important to monitor their properties over time. We did not observe aggregation of any of the formulations and all liposomes had a diameter around 100 nm.

### Binding of OX26MAb-/LB509MAb-PEGylated liposomes to target proteins

The ability of the antibody-decorated liposomes to continue to recognize their ligands was assessed by ELISA. First, each ligand (transferrin receptor, α-synuclein or PBS as a control) was adsorbed to the bottom surface of 96-well plates, and then incubated with the respective immunoliposomes (formulations B, C and D) or with PEGylated liposomes (A) as control; finally, to assess the formation of the ligand-antibody binding, a secondary antibody was used to reveal the presence or absence of immunoliposomes. Control liposomes (A) showed an absorbance at 405 nm of around 0.30 in all the wells with different types of coating (Figure 3). Immunoliposomes with LB509MAb (liposomes B) gave an increase in the absorbance in the wells coated with α-synuclein (0.73 ± 0.04). Immunoliposomes coupled to OX26MAb (liposomes C) had higher absorbance in the wells coated with transferrin receptor (0.69 ± 0.03). Importantly, the immunoliposomes coupled to both MAbs (liposomes D) showed functionality for both ligands as concluded by the absorbance at 405 nm of 0.71 ± 0.04 and 0.70 ± 0.02, when testing against α-synuclein and transferrin receptor, respectively (Figure 3). We did not observe nonspecific binding of immunoliposomes to α-synuclein or transferrin receptor. The MAbs are unmodified and thus they interact with a single target, as they are specific for a single epitope of an antigen. The OX26MAb-/LB509MAb-PEGylated liposomes show similar activity in terms of binding to the two different targets, transferrin receptor and α-synuclein.

### Catechin release from OX26MAb-/LB509MAb-PEGylated liposomes

The encapsulation efficiency of EGCG was about 76 ± 7%. The size, solubility, composition and biodegradation of the liposome are the main factors that affect the release rate of a drug [17]. Besides, a time compromise for the drug release profile is needed. If the catechin-loaded liposomes are too stable, the drug release will not occur but if the drug is not well entrapped, it will leak prematurely. We studied the release profile of EGCG from immunoliposomes (with the two antibodies) using the dialysis method in PBS (pH 7.4) at 4 and 37°C. The release profile of the catechin from the immunoliposomes showed a regular pattern with a continuous release. At 37°C, EGCG was released from the liposomes during the first 12 h in a percentage of approximately 25% (Figure 4). After 44 h, the release of EGCG reached a percentage of cumulative release of about 40%, indicating a sustained release. The presence of the two antibodies on the surface of the PEGylated liposomes does not prevent the release of the tested drug.

### *In vitro* uptake & cytotoxicity of OX26MAb-/LB509MAb-PEGylated liposomes

The immortalized hCMEC/D3 cell line has been demonstrated to mimic the endogenous microvascular brain endothelial cells due to expression of tight junction proteins [18–21]. The cells were incubated with the liposomes for 2 and 6 h at 37°C. The relative uptake efficiency corresponds to the amount of liposomes in cells divided by the initial concentration of liposomes.

The uptake of control liposomes (PEGylated liposomes with no antibody attached – formulation A) and liposomes with anti-α-synuclein antibody (formulation B) by hCMEC/D3 at concentrations of 60 µM is comparatively much lower than the uptake of liposomes with OX26MAb (C) and liposomes with the two antibodies (D) (Figure 5). The uptake ratios at 60 µM were 5.9% for formulation A, 4.3% for B, 10% for C and 13% for D. The results indicate that OX26MAb increases the uptake efficiency of liposomes by the BBB cell model. As the immunoliposome concentration increases, the cellular uptake percentage decreases, which indicates a saturable uptake mechanism. The cellular uptake of liposomes after 6 h incubation time at 37°C shows similar trend as the incubation for 2 h.

The cytotoxicity of the liposomes was assessed by using the Alamar Blue assay, which measures quantitatively the proliferation of cell lines. The results demonstrate that all different liposome formulations in the concentration range of 60–200 µM are not toxic to hCMEC/D3 cells (Figure 6). The safety of nanocarriers is crucial for their use in clinics and should be considered in all steps of drug development studies. Previous studies report that the weekly administration of PEGylated immunoliposomes to rats does not cause toxic effects, no inflammation in the brain [22].

The results of this study support the following conclusions. The conjugation of the MAb against the α-synuclein to OX26MAb-decorated liposomes does not alter their cellular uptake by the BBB cell model hCMEC/D3. The OX26MAb-/LB509MAb-PEGylated liposomes show similar cellular uptake efficiency as OX26MAb-PEGylated liposomes. The cellular uptake efficiency (ratio of internal fluorescence compared with total fluorescence) decreases with the increasing of the concentration of immunoliposomes, which might indicate a saturable uptake mechanism. Increasing the incubation time has little effect on the cellular uptake efficiency at any of the concentrations tested, likely due to exocytosis of the immunoliposomes.

The presence of receptor-mediated transport (RMT) system within the BBB, such as the transferrin receptor, enables the use of an endogenous peptide or peptidomimetic MAb that is able to cross the BBB via a specific RMT system (molecular Trojan horse technology) [23]. MAbs are preferable over the endogenous peptides because the plasma concentration of the latter is high leading to a saturation of the protein binding sites on the BBB receptor. MAbs bind to epitopes on the BBB receptor that are removed from the endogenous ligand. The conjugation of PEGylated liposomes to antibodies against the transferrin receptor for *in vivo* brain targeting of drugs was proposed almost two decades ago [24]. An important property of these systems is their ability to transcytose through the BBB. The MAb against transferrin receptors used here, the OX26MAb to the rat transferrin receptor, was shown to traverse the BBB by a similar mechanism that mediates the BBB transcytosis of endogenous transferrin [25,26]. Importantly, OX26MAb-decorated liposomes were found to undergo transcytosis across monolayers of immortalized RBE4 rat brain endothelial cells [27]. Another study had established that the uptake and transcytosis of liposomes functionalized with OX26MAb by hCMEC/D3 cells are higher than those of the liposomes without MAb [28]. The study had also indicated that the uptake mechanism of OX26MAb-decorated liposomes is receptor-mediated.

The development of liposomes with dual-targeting properties enables noninvasive delivery of drugs to areas of the brain affected by a particular disease [8]. One ligand enables the transport of liposomes through the BBB and a second ligand allows the binding of the system to the drug target. This strategy is currently being explored in Alzheimer's disease research studies [29–31]. Immunoliposomes with two different ligands, one to target the BBB and a second to target the amyloid beta-peptide (main component of Alzheimer's disease plaques), have been reported [14,32–33]. PEGylated immunoliposomes doubly targeted with MAbs had also been reported to deliver RNAi expression plasmid for the treatment of brain cancer [34]. This field needs more active research and developments in order to obtain optimized systems.

## Conclusion

The present study demonstrates that OX26MAb-/LB509MAb-PEGylated liposomes have affinity for both the ligands transferrin receptor and α-synuclein (neuronal protein that is linked to Parkinson's disease) and that they are internalized by immortalized human cerebral microvascular endothelial cells. The drugs are encapsulated in the liposomes and the surface of the liposome is coated with PEG, which restricts the liposome uptake by the reticuloendothelial system. The receptor-specific MAbs can then target the liposome across biological membranes.

## Future perspective

Targeting nanocarriers to the brain with only one type of mAbs that target a singular antigen has limitations. Noninvasive delivery of drugs to the brain requires both their transcytosis through the BBB and their transport to the target site. At least two ligands are necessary for efficient drug targeting to the brain. The advances in this field will probably lead to even more complex systems with several ligands to target multicomponents. Liposomes are already in clinical use for the treatment of cancer and infectious diseases and the tendency will be to develop further the liposome technology to deliver therapeutics to the brain in neurodegenerative diseases. More studies are necessary to determine whether this strategy will become a standardized way of delivering drugs to the brain.

**Table 1.** **Mean diameter, polydispersity index and zeta potential of immunoliposomes.**

**Liposome formulation**	**Mean diameter (nm)**	**PDI**	**Zeta potential (mV)**
PEGylated liposomes	86 ± 1	0.14 ± 0.01	0.2 ± 0.4
LB 509MAb-PEGylated liposomes	98 ± 3	0.17 ± 0.01	-1.8 ± 0.2
OX26MAb-PEGylated liposomes	104 ± 4	0.19 ± 0.01	-1.5 ± 0.1
OX26MAb-/LB509MAb-PEGylated liposomes	105 ± 6	0.21 ± 0.01	-2.8 ± 0.3

Values given as mean ± standard deviation.

The tip of some of the PEG molecules was conjugated to anti-α-synuclein monoclonal antibody (LB 509MAb), anti-transferrin receptor antibody (OX26MAb) or to both antibodies. Error bars represent standard deviations over three replicas.

PDI: Polydispersity index; PEG: Polyethylene glycol.

**Executive summary**

Immunoliposomes targeted to rat transferrin receptor with OX26 monoclonal antibody and to α-synuclein with LB509 monoclonal antibody are prepared.
The doubly targeted immunoliposomes react with transferrin receptors and to α-synuclein with the same efficiency as the liposomes conjugated to only the respective single antibody.
The cellular uptake efficiency of doubly targeted immunoliposomes by a model of the endothelial cells of the blood–brain barrier (hCMEC/D3 cell line) is comparable to the liposomes targeted only to transferrin receptors.
The immunoliposomes do not show cytotoxicity at the concentrations tested (up to 200 μM).
The increase of the immunoliposome concentration in the cellular uptake studies indicates a saturable mechanism, which is consistent with a receptor-mediated uptake mechanism.
